# Ku80 promotes melanoma growth and regulates antitumor effect of melatonin by targeting HIF1-α dependent PDK-1 signaling pathway

**DOI:** 10.1016/j.redox.2019.101197

**Published:** 2019-04-20

**Authors:** Tianze Liu, Lizi Jin, Miao Chen, Zongheng Zheng, Wenjing Lu, Wenhua Fan, Liren Li, Fufu Zheng, Qiaohua Zhu, Huijuan Qiu, Jiani Liu, Manyu Chen, Chunfang Tian, Zheng Hu, Changlin Zhang, Meihua Luo, Jian Li, Tiebang Kang, Lukun Yang, Yizhuo Li, Wuguo Deng

**Affiliations:** a Sun Yat-sen University Cancer Center, State Key Laboratory of Oncology in South China, Collaborative Innovation Center of Cancer Medicine, Guangzhou, China; b The Third Affiliated Hospital, Sun Yat-sen University, Guangzhou, China; c The First Affiliated Hospital, Sun Yat-sen University, Guangzhou, China; d Shunde Hospital, Southern Medical University, Foshan, China; e Institute of Cancer Stem Cell, Dalian Medical University, Dalian, China; f The Fifth Affiliated Hospital, Sun Yat-sen University, Zhuhai, Guangdong, China

**Keywords:** Ku80, PDK-1, Melatonin, HIF1-α, Melanoma

## Abstract

Melanoma is one of the most malignant and aggressive cancers with high cancer-related deaths. However, it is unclear whether Ku80 regulates tumor growth in human melanoma. In this study, we screened a siRNA library targeting 6024 human genes and identified Ku80 as a potential therapeutic target in melanoma cells. Knockdown of Ku80 significantly suppressed melanoma cell proliferation and induced apoptosis, as well as enhanced the antitumor effect of melatonin in melanoma *in vitro* and *in vivo.* Overexpression of Ku80, however, promoted melanoma growth and increased the insensitivity of melanoma cells to melatonin. Mechanistically, we found that Ku80 bound to the PDK1 promoter and activated the transcription of PDK1. Moreover, we showed that the binding of Ku80 at the PDK-1 promoter was HIF1-α dependent, and melatonin degraded HIF1-α in melanoma cells. Furthermore, clinical data revealed that the expression of Ku80 and PDK-1 proteins were positively correlated and elevated in the tumor tissues of melanoma patients, and high expression of Ku80 predicted a poor prognosis in melanoma. Collectively, our study demonstrated that Ku80 promoted melanoma growth and regulated antitumor activity of melatonin by targeting HIF1-α dependent PDK-1 signaling pathway, suggesting that Ku80 may be a potential molecular target for melanoma treatment.

## 1 Introduction

Melanoma is one of the leading causes of death from skin cancer, and its incidence is increasing globally [1]. Due to the aggressiveness of melanoma, patients with melanoma are often diagnosed at advanced stages with local or even remote metastasis, when it is incurable by surgery only. Besides, melanoma is one of the most drug-resistant human cancers [2]. Accordingly, patients with metastatic melanoma had a median survival time of less than 1 year [3], The long term survival rate for patients with metastatic melanoma is only 5% [4]. Since 2011, treatment of melanoma has been revolutionized as a result of better understanding of its biology and tumor immunology [5]. As half of the advanced melanoma patients bear mutations at the residue Val 600 in the kinase BRAF that lead to constitutive activation of the MAPK pathway [6], targeted therapies including BRAF inhibitors (vemurafenib, dabrafenib) and MEK inhibitors (trametinib, cobimetinib) have been developed [[7], [8], [9], [10], [11]]. While BRAF inhibitors have improved the overall response rates and survival compared to chemotherapy [11], their effect is short-term in most patients due to the development of resistance at a median of 5–7 months [5], even though their combination with MEK inhibitors has demonstrated benefit in progression free survival [12,13]. Immunotherapy has been another revolutionary treatment for melanoma. Monoclonal antibodies against CTLA-4 (ipilimumab) and PD-1 (pembrolizumab, nivolumab) to block the tumor-induced immune checkpoints have successfully prolonged the overall survival of patients with advanced melanoma [[14], [15], [16]]. At the same time, it should be noted that checkpoint blockade can sometimes lead to serious adverse events due to nonspecific immunologic activation [17]. For instance, ipilimumab was reported to have autoimmune toxicities associated with CTLA-4 blockade [18], concurrent administration of vemurafenib and ipilimumab caused unacceptable hepatotoxicity [19]. Therefore, it is of importance to further understand the pathogenesis of melanoma, and to discover novel molecular targets for the development of effective therapies with lower toxicity.

Ku80 is a subunit of the conserved DNA binding heterodimer Ku70/Ku80, which plays a critical role in guarding chromosome stability by repairing DNA double strand breaks via the non-homologous end joining (NHEJ) pathway [20]. Besides, the Ku heterodimer functions in many nuclear processes, which include telomere maintenance [21], V(D)J recombination [22], cell cycle regulation [23], and transcription regulation [24,25]. Ku80 is often deregulated in tumor samples, and its expression can be a prognostic factor. For example, constitutive activation of NF-κB and expression of COX-2 enhanced Ku expression and its DNA end-binding activity, leading to gastric cell proliferation and tumorigenesis [26]. Consistently, Ku was found to have higher association with chromatin in metastatic breast cancer cells than in hyperplastic and normal breast cells, and replication origins like the *c-myc* origin were more active as a result of this increased association [27]. Moreover, Harima Y found that high expression of Ku80 correlated with decreased tumor radiosensitivity in patients with cervical cancer [28]. And Komuro Y found that the expression pattern of Ku correlates with tumor radiosensitivity and disease free survival in patients with rectal carcinoma [29]. Similarly, Saviozzi S found that transcriptional overexpression of Ku80 was associated with a poor prognosis in non-small cell lung cancer patients [30]. In non-melanoma skin cancer, Ku80 protein level was significantly increased in basal and squamous cell carcinomas, and positively correlated to DNA-binding activity as well as cell proliferation rate [31]. In melanoma, high expression of Ku80 was related to significantly worse survival [32,33]. Nonetheless, it remains unclear whether and how Ku80 regulates the growth and sensitivity of cells to chemotherapy in melanoma.

Melatonin (*N*-acetyl-5-methoxytryptamine), an indoleamine compound produced in the pineal gland and secreted into circulating blood. Melatonin has a wide range of reported biologic effects including circadian rhythm, sleep, mood, sexual maturation, stress, insulin secretion, and reproduction [34]. As a potent reactive oxygen species scavenger, melatonin plays an important role in various cardiovascular diseases, including myocardial ischemia-reperfusion injury [35], atherosclerosis [36], hypertension [37], heart failure [38]. Recent experimental research of melatonin highlights its antitumor effects through its antioxidant effect, induction of apoptosis and cell cycle arrest, inhibition of metastasis, and suppression of angiogenesis. It possesses chemotherapeutic potential in human cancers such as hepatocellular carcinoma and glioblastoma [39,40]. However, the precise therapeutic effects and underlying mechanisms of action of melatonin in melanoma are still not so clear.

In this study, we screened a siRNA library in A375 cells to discover potential therapeutic targets for melanoma, and found that knockdown of Ku80 inhibited cell growth and induced apoptosis in melanoma cells. In contrast, overexpression of Ku80 remarkably promoted the growth of melanoma cells. Global transcriptome analysis revealed that PDK1 was a target gene regulated by Ku80. Mechanistically, Ku80 bound to the promoter of PDK1 to enhance its transcription, and overexpression of PDK1 partially reversed the growth inhibition caused by Ku80 knockdown in melanoma cells and mouse xenograft models. Further study showed Ku80 regulated PDK1 transcription was HIF1-α dependent. Additionally, we demonstrated that the melatonin exerted its antitumor effect via HIF1-α mediated Ku80/PDK1 pathway in part, and downregulation of Ku80 enhanced the antitumor effect of melatonin in melanoma. Inversely, overexpression of Ku80 partially reverted the antitumor effect of melatonin. We also proved that melatonin synergize with Ku80 knockdown to inhibit cells growth through HIF1-α dependent Ku80/PDK1 pathway. Finally, our clinical data indicated that the expression of Ku80 and PDK1 were positively correlated in melanoma tissues, and their elevated expression was associated with poor prognosis in melanoma patients. Collectively, our study has elucidated that Ku80 interacted with HIF1-α regulated melanoma growth and antitumor effect of melatonin through enhancing the expression of PDK1, suggesting that Ku80 may be a potential target and melatonin combined with Ku80 inhibitor may be a more powerful treatment mode for melanoma treatment.

## 2 Material and methods

### 2.1 Cell lines and cell culture

Human melanoma cell lines A375, MEWO, A431, Sk-mel-28 and WM35 were obtained from American Type Culture Collection (ATCC, Manassas, VA), cultured in Dulbecco's Modified Eagle Medium (DMEM, Invitrogen, Carlsbad, CA) supplemented with 10% fetal bovine serum (FBS), 100 μg/ml penicillin and 100 μg/ml streptomycin, and maintained in a humidified atmosphere with 5% CO_2_ at 37 °C.

### 2.2 Antibodies and reagents

IgG (#2729) and antibodies for Ku80 (#2753), PDK1 (#3820), HIF-1α (#3716), Histone H3 (#4499), GAPDH (#5174), PI3K (#4255), p-PI3K (#4228), AKT (#4691), *p*-AKT (#4060), ERK (#9102), *p*-ERK (#9102), mTOR (#2972), *p*-mTOR (#2971), MMP2 (#4022), MMP9 (#3852), PARP (#9542), cleaved-caspase 3 (#9661) and cleaved-caspase 9 (#9505) were purchased from Cell Signaling Technology (Danvers, MA). Melatonin (S1204) was purchased from Selleck Chemicals (Houston, TX), dissolved in DMSO, and added into cell culture medium for a final concentration of 1 mM.

### 2.3 Cell viability assay

Cells were seeded at a density of 3 × 10^3^ cells per well in 96-well plates, and the viability of the cells was assessed by the MTS assay (Promega, Madison, WI) 48 h later. The absorbance value at 490 nm in each well was measured with a microplate reader. All experiments were performed in 6 replicates per trial, with 3 independent trials in total and the average percentages of cell viability were shown.

### 2.4 Colony formation assay

Cells were seeded at a density of 200–400 cells per well in 6-well plates and cultured for 2 weeks. The colonies were then stained with 1% crystal violet and counted. All experiments were performed in 3 independent trials.

### 2.5 Western blot

Whole cell lysates or nuclear extracts were prepared using Complete Lysis-M reagent (Roche, Indianapolis, IN) and RIPA lysis buffer (Beyotime Biotechnology, Shanghai, China). Protein concentration was determined by BCA assay (ThermoFisher Scientific, Waltham, MA). The proteins were separated in 8%–10% SDS-PAGE gels and transferred onto PVDF membranes for detection.

### 2.6 Apoptosis assay

Cells (1 × 10^5^ cells/ml) were stained with 5 ml AnnexinV-FITC and 5 ml PI (propidium iodide), incubated in room temperature for 15 min in the dark, and then analyzed by flow cytometry (EPICS XL, Beckman Coulter, Brea, CA). Apoptosis was determined as FITC-positive in cells. Cell cycle analysis was performed using PI staining.

### 2.7 RT-qPCR

Total RNA was isolated using TRIZOL Reagent (Invitrogen, Carlsbad, CA), and cDNA was synthesized using the ReverTra Ace qPCR RT Master Mix (Toyobo, Osaka, Japan). The SYBR Green PCR master mix (Toyobo) was then used for qPCR, which was followed by detection with a Bio-Rad CFX96 and analyzed with the Bio-Rad Manager software (Bio-Rad, Hercules, CA). Each sample was tested in triplicate.

### 2.8 Streptavidin-agarose pulldown assay

The PDK1 promoter binding proteins were analyzed by streptavidin-agarose pulldown assay. Briefly, 1 mg of nuclear protein extracts from human melanoma cells were incubated with 10 μg of biotin-labeled double-stranded DNA probes corresponding to nucleotide −875 ∼ +40 of the PDK1 promoter region (Sigma-Aldrich, St Louis, MO) and 100 μl of streptavidin-agarose beads (Sigma-Aldrich) at 4 °C over night. The mixture was then centrifuged at 500×*g* to pulldown the DNA-protein complex.

### 2.9 Chromatin immunoprecipitation (ChIP)

Cells were fixed with 1% formaldehyde, and the cross-linking was quenched by adding in 1.375 M glycine (100 μl/ml of culture). The samples were sonicated on ice to shear the DNA into 300 to 1000 bp fragments. For each total cell lysate, one third was used as the DNA input control, another third was immunoprecipitated with Ku80 antibody, and the last third was subjected to non-immune rabbit IgG. DNA fragments were purified by spin columns (Qiagen, Hilden, Germany), and PCR was performed to amplify the promoter region of PDK1 with the following primer pair - Forward: 5′-ACG CAG ATT GGT GGT TC-3′, Reverse: 5′-AGA GAA GCC ACA GCC AGT-3’. The PCR products were resolved by electrophoresis in a 2% agarose gel and visualized by Gel-Red staining.

### 2.10 Promoter reporters and dual-luciferase assay

A fragment containing the promoter region of PDK1 (−875 ∼ +40) was inserted between the *Sac*I and HindІІІ sites of the firefly luciferase vector pGL4.10 (Promega, Madison, WI), and Renilla luciferase reporter vector pRL-TK was used as a control. Cells (2 × 10^4^ cells/well) were seeded in a 96 well plate. After the cell confluence reached 50%, pGL4.10-hTERT-387 or pRL-TK was transfected into melanoma cells with Ku80 stable knockdown or overexpression with Lipofectamine 3000 (Invitrogen, Carlsbad, CA) (30:1 ratio). 72 h after transfection, dual-luciferase assay was performed using the Dual-Luciferase® Reporter Assay System (Promega, Madison, WI).

### 2.11 Confocal immunofluorescence

For confocal microscopy analysis, cells grown on chamber slides were washed with PBS, fixed with paraformaldehyde and permeabilized with pre-cooled methyl alcohol for 15 min at −20 °C. The samples were then pretreated with 10% bovine serum albumin (BSA) in PBS for 30 min, and specific antibodies were added and incubated overnight at 4 °C. Following five 5 min washes with PBS, secondary antibodies were added and incubated for another hour. After five additional 5 min washes, samples were examined using the confocal microscope.

### 2.12 Co-immunoprecipitation assays

Equal amounts of nuclei protein extracts prepared from different cell lines were incubated with the indicated antibodies. Then, the agarose-conjugated protein-A/G beads (Merck Millipore, Billerica, MA) were added and the mixture was incubated at 4 °C overnight. After extensive washing with cold phosphate-buffered saline (PBS), the beads were mixed with loading buffer and boiled. The proteins in the supernatant were detected by Western blotting analysis.

### 2.13 Animal experiment

All animal procedures were performed in accordance with the Guide for the Care and Use of Laboratory Animals (NIH publications Nos. 80-23, revised 1996) and the Institutional Ethical Guidelines for Animal Experiments developed by Sun Yat-sen University. Female athymic nude mice aged with 3- to 4-weeks were purchased and housed in the animal center of Sun Yat-sen University. A375 cells with control shRNA, Ku80 shRNA, vector, Ku80 or PDK1 overexpression plasmid were injected (5 × 10^6^ cells in 100 μl PBS) subcutaneously into the left flank of each mouse. When the formed tumor reached 100 mm^3^, the animals were randomly divided into groups of 5. For melatonin treatment, mice were intratumorally injected with melatonin (25 mg/kg) every other day for 3 weeks, while the control group received PBS. The tumor size was measured using vernier calipers once every three days and volumes were calculated as V = (length × width × height)/2. At the end of the experiment, the mice were sacrificed, and the tumors were excised, weighed and processed for immunohistochemical analyses.

### 2.14 Tissue microarrays

The tissue microarray for analysis of Ku80 and PDK1 protein expression in melanoma and normal tissues was purchased from Fanpu Biotech, Inc (Guilin, China). Detailed clinical information, including age, gender and location of the melanoma, was available for all cases. For prognosis analysis, the melanoma tissue microarray with overall survival information of each case was purchased from Novus Biologicals (Littleton, CO).

### 2.15 Immunohistochemical (IHC) assay

Briefly, tissue sections were deparaffinized, rehydrated and then immersed in target retrieval solution (pH 6; Dako Cytomation, Hamburg, Germany) and boiled at medium baking temperature for 20 min once in a microwave. After blocking the slides with 3% BSA, the sections were then incubated with primary antibody against Ku80 (1:100) and PDK1 (1:50). Then, the sections were incubated with rabbit and mouse conjugated-second antibody for 45 min at 37 °C and the specimens were counterstained with hematoxylin and the target-positive cells were counted in 3–4 different fields and photographed using CellSens Dimension (version 1.8.1, Olympus). The staining intensity was measured with ImagePro Plus (version 6.0, Media Cybernetics).

### 2.16 Statistical analysis

Data were presented as mean ± standard deviation from at least three independent experiments. Statistical analysis was carried out using SPSS 11.0 software (SPSS Inc.; Chicago, IL). *P* < 0.05 was considered statistically significant.

## 3 Results

### 3.1 Ku80 promotes proliferation and inhibits apoptosis in melanoma cells

**Fig. 1:**
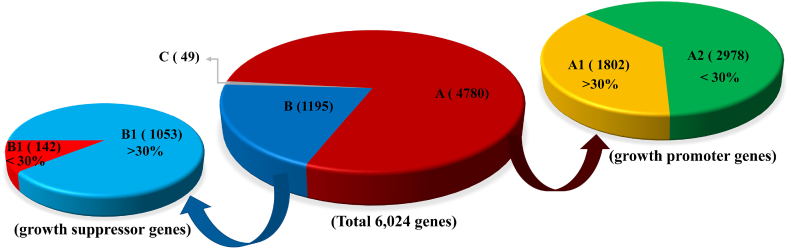
**Inhibition of cell viability by siRNA library targeting the 6024 human genes. (A)** Genes whose knockdown inhibited cell viability. **(B)** Genes whose knockdown promoted cell viability. **(C)** Genes whose knockdown had nothing to do with cell viability. **(A1)** Genes whose knockdown inhibited cell viability with the inhibition ratio more than 30%. **(A2)** Genes whose knockdown inhibited cell viability with the inhibition ratio less than 30%. **(B1**) Genes whose knockdown promoted cell viability with the promotion ratio more than 30%. **(B2)** Genes whose knockdown promoted cell viability with the promotion ratio less than 30%.

**Fig. 2:**
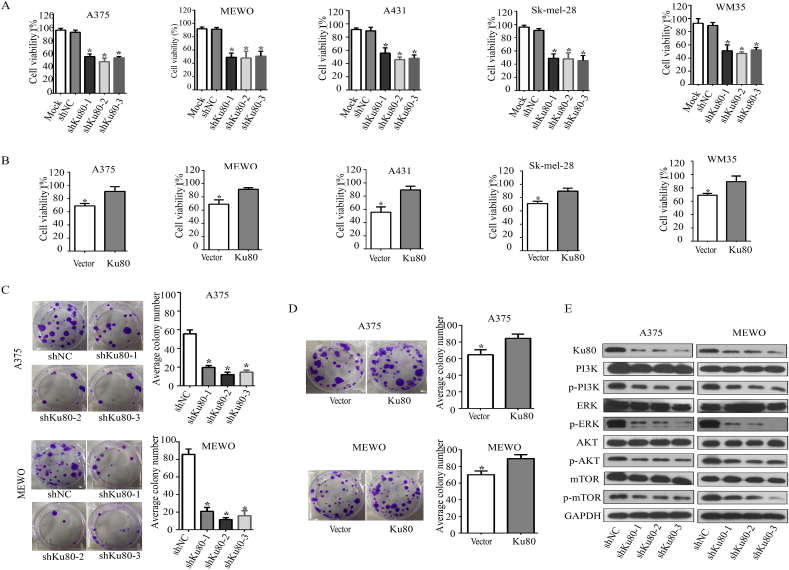
**Knockdown of Ku80 inhibited melanoma cell growth. (A)** A375, MEWO, A431, Sk-mel-28 and WM35 melanoma cells were transfected with Ku80-specific small hairpin RNAs (shKu80-1, shKu80-2 and shKu80-3) or control shRNA (shNC). After 48 h, cell viability was measured by MTS assay. **(B)** A375, MEWO, WM35 and Sk-mel-28 melanoma cells were transfected with Ku80 overexpressing plasmid (Ku80) or control vector (Vector), and cell viability was measured by MTS assay after 48 h. **(C)** A375 and MEWO cells were transfected with Ku80 shRNAs or control shRNA. 14 days later, formed colonies were stained with crystal violet and counted. **(D)** A375 and MEWO cells were transfected with Ku80 overexpressing plasmid or vector, and after 14 days, colonies were stained and counted. **(E)** The expression of Ku80, as well as the total and phosphorylated PI3K, ERK, Akt and mTOR were detected by Western blot in A375 and MEWO cells 48 h after shRNA transfection. The experiments were done in triplicates, and the results were presented as mean ± SD, with *P < 0.05 by student's *t-*test.

In order to discover proteins involved in the growth of melanoma, we screened a siRNA library targeting >6000 genes in human melanoma A375 cells, and found that 4780 genes whose knockdown inhibited cell viability (Fig. 1). Of the 4780 genes, 1802 genes whose knockdown inhibited cell viability with the inhibition ratio more than 30% (Fig. 1, A1). In addition 1195 genes whose knockdown promoted cell viability (Fig. 1, B). Ku80 was one of genes whose knockdown significantly suppressed the cell viability. To validate that Ku80 could promote melanoma cell growth, we first measured its effect on the viability of a panel of human melanoma cell lines (A375, MEWO, A431, Sk-mel-28 and WM35) by MTS assay. As shown in Fig. 2A, knockdown of Ku80 by its specific shRNAs dramatically inhibited the viability of all tested cell lines. In contrast, overexpression of Ku80 significantly enhanced the viability of melanoma cells (Fig. 2B). Next, colony formation assay was performed and showed that knockdown of Ku80 remarkably decreased the colony formation capacity of melanoma cells (Fig. 2C), whereas overexpression of Ku80 increased their colony formation capacity (Fig. 2D). To elucidate the molecular mechanism by which Ku80 regulated melanoma cell proliferation, we examined the expression and phosphorylation of key proteins in growth regulation, and found that knockdown of Ku80 repressed the phosphorylation of mTOR, PI3K, Akt and ERK (Fig. 2E).

**Fig. 3:**
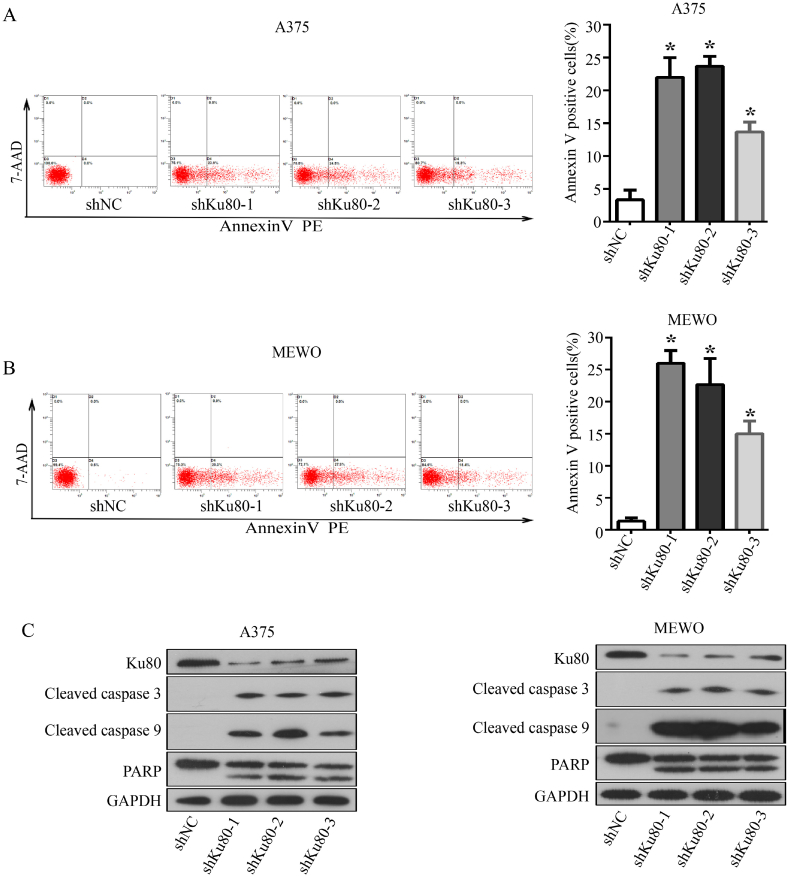
**Knockdown of Ku80 increased apoptosis of melanoma cells. (A**–**B)** A375 **(A)** and MEWO **(B)** cells were transfected with Ku80 shRNAs (shKu80-1, shKu80-2 and shKu80-3) or control shRNA (shNC). 72 h later, the cells were stained with Annexin V-FITC/PI, and their apoptosis was determined by FACS analysis. **(C)** 72 h after shRNA transfection, the expression of Ku80, cleaved-caspase 3, cleaved-caspase 9 and PARP proteins in A375 and MEWO cells were detected by Western blot. The experiments were done in triplicates, and the results were presented as mean ± SD, with *P < 0.05 by student's *t-*test.

We then investigated whether Ku80 inhibited apoptosis of melanoma cells. FACS analysis revealed that A375 and MEWO cells with Ku80 knockdown had a higher apoptosis rate than the control cells (Fig. 3A and B). Consistently, Western blot showed that cleaved caspase 3 and cleaved caspase 9 were increased while PARP was decreased, when Ku80 was knocked down in A375 and MEWO cells (Fig. 3C). Collectively, our results indicated that Ku80 inhibited apoptosis of melanoma cells, and promoted their proliferation through mTOR/Akt signaling pathways.

### 3.2 Ku80 binds to the PDK1 promoter and regulates the promoter activity and expression of PDK1

**Fig. 4:**
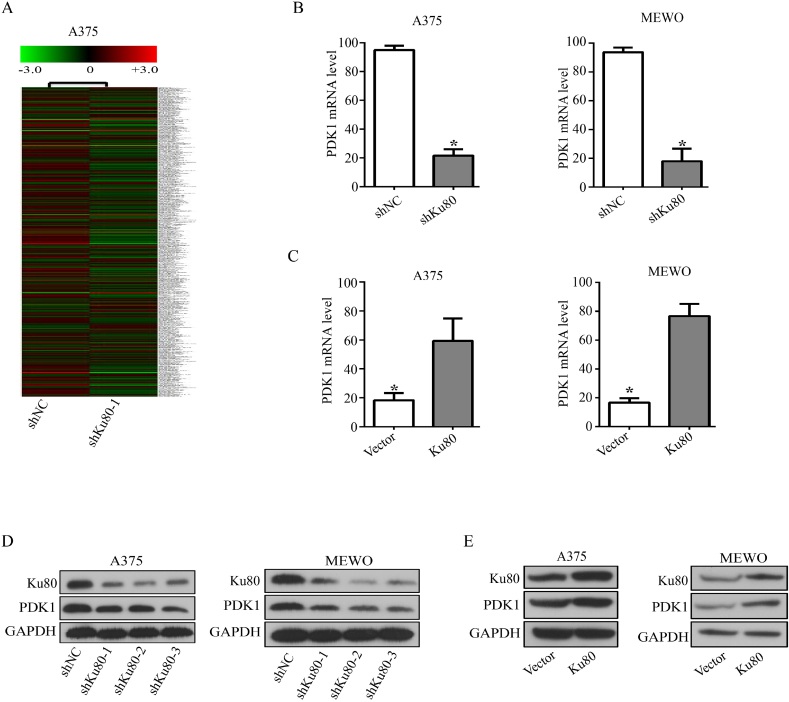
**Ku80 regulated the promoter activity and expression of PDK1**. **(A)** Heat maps from global comparative transcriptome analysis. A375 cells were transfected with Ku80 shRNA (shKu80-1) or control shRNA (shNC). 48 h later, total RNA was extracted from the cells for analysis. **(B**–**E)** Ku80 regulated the expression of PDK1 in melanoma cells. RT-qPCR was used to measure the relative level of PDK1 mRNA in A375 and MEWO cells transfected with Ku80 shRNA (B) or Ku80 overexpression plasmid **(C)**. Western blot was used to detect the protein level of PDK1 in A375 and MEWO cells transfected with Ku80 shRNA **(D)** or Ku80 overexpression plasmid **(E)**. The results were presented as mean ± SD of three independent trials, with *P < 0.05 by student's *t-*test.

**Fig. 5:**
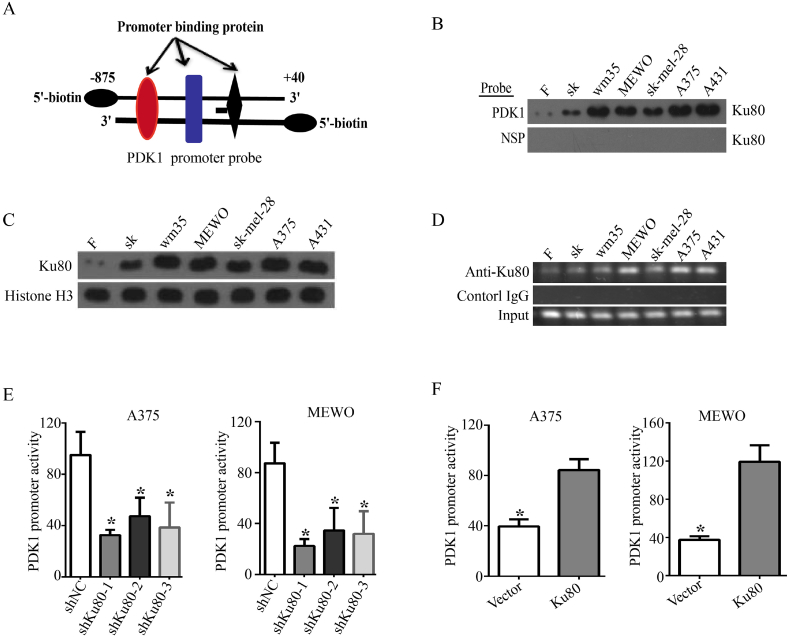
**Ku80 bound to the PDK1 promoter. (A)** A cartoon showing the design of the 5′-biotin labeled double-stranded PDK1 promoter probe (−875 ∼ +40). **(B)**. The nuclear protein extracts from human immortalized fibroblasts **(F)**, immortalized skin cells (SK) and five different melanoma cell lines (WM35, MEWO, Sk-mel-28, A375 and A431) were incubated with the PDK1 promoter probe (A) to pull down the proteins bound on it. Binding of Ku80 on the PDK1 promoter probe or a nonspecific probe (NSP) was detected by Western blot using Ku80 specific antibody. **(C)** The expression of nuclear Ku80 in the cells in **(B)** was detected by Western blot, and histone 3 was used as a loading control. **(D)** ChIP with Ku80 specific antibody was performed in the cells in **(B)**. The precipitated PDK1 promoter fragments were amplified by PCR using PDK1 promoter specific primers, and the PCR products were separated in 2% agarose gels. IgG was used as a negative control. **(E)** A375 and MEWO cells were transfected with Ku80 shRNAs (shKu80-1, shKu80-2 and shKu80-3) or control shRNA (shNC). After 72 h, relative PDK1 promoter activity was determined by dual-luciferase assay. **(F)** A375 and MEWO cells were transfected with Ku80 overexpressing plasmid (Ku80) or control vector (Vector). 72 h later, dual-luciferase assay was performed to determine relative PDK1 promoter activity. The results were presented as mean ± SD of three independent trials, with *P < 0.05 by student's *t-*test.

To further clarify the downstream proteins that mediated the effects of Ku80 in melanoma cells, we established Ku80 knockdown and control stable cell lines, and performed transcriptome analysis. The result showed that PDK1 (pyruvate dehydrogenase kinase 1) was one of the candidates regulated by Ku80, as its expression was dramatically decreased in Ku80 knockdown cells (Fig. 4A). RT-qPCR validated that the mRNA level of PDK1 was significantly decreased when Ku80 was knocked down in A375 and MEWO cells (Fig. 4B), and it was increased remarkably in Ku80 overexpressing A375 and MEWO cells (Fig. 4C). Consistently, the protein level of PDK1 was decreased in A375 and MEWO cells with Ku80 knockdown (Fig. 4D), but increased in cells with Ku80 overexpression (Fig. 5E).

Considering that Ku80 regulated the expression of PDK1 on the mRNA level, we deduced that Ku80 might bind at the promoter region of PDK1 to activate its transcription. To test this hypothesis, we designed a biotin-labeled double-stranded DNA probe that contained the sequence of the −875 ∼ +40 region of the PDK1 gene promoter (Fig. 5A), and incubated it with the nuclear proteins from human immortalized fibroblasts (F), immortalized skin cells (SK) and five different melanoma cell lines (WM35, MEWO, Sk-mel-28, A375 and A431). We then used streptavidin-agarose beads to pull down the proteins bound to the biotin-labeled probe, and separated them by SDS-PAGE. Western blot showed that Ku80 was specifically detected among the nuclear proteins bound at the PDK1 promoter (Fig. 5B), and it was more enriched in the nuclei of melanoma cells (Fig. 5C). Moreover, we examined the binding of Ku80 at the endogenous promoter of PDK1 by ChIP, and confirmed that Ku80 had increased association with the PDK1 promoter in melanoma cells (Fig. 5D). In addition, we inserted a fragment containing the promoter region of PDK1 into the firefly luciferase vector, and used the *Renilla* luciferase reporter vector as internal control for dual luciferase reporter assay. The results showed that knockdown of Ku80 significantly decreased the PDK1 promoter activity in A375 and MEWO cells (Fig. 5E), whereas overexpression of Ku80 dramatically enhanced the PDK1 promoter activity in A375 and MEWO cells (Fig. 5F). Collectively, our experiments revealed that Ku80 regulated the promoter activity and transcription of PDK1.

### 3.3 Ku80 regulates melanoma growth through PDK1 pathway *in vitro* and *in vivo*

**Fig. 6:**
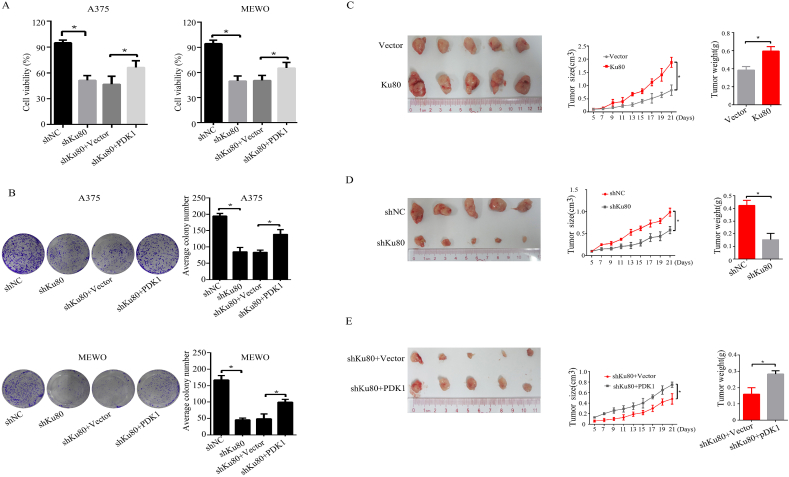
**Ku80 regulated melanoma growth through PDK1 pathway *in vitro* and *in vivo***. **(A**–**B)** A375 and MEWO cells were transfected with control shRNA (shNC), Ku80 shRNA (shKu80), Ku80 shRNA + control vector (shKu80+Vector), and Ku80 shRNA + PDK1 overexpression plasmid (shKu80+PDK1) respectively. After 48 h, the cell viability was measured by MTS assay **(A)**, and the colony formation capacity was determined by colony formation assay **(B)**. **(C**–**E)** A375 stable cells with control vector (Vector), Ku80 overexpression plasmid (Ku80), control shRNA (shNC), Ku80 shRNA (shKu80), Ku80 shRNA + control vector (shKu80+Vector), or Ku80 shRNA+PDK1 overexpression plasmid (shKu80+PDK1) were subcutaneously injected into the flank of the nude mice. Tumor size was recorded every 2 days over 3 weeks (middle panels), and at the end of the experiment, the xenografts were excised after the mice were humanely sacrificed. Tumor volume was then captured (left panels), and tumor weight was measured (right panels). The results were presented as mean ± SD (n = 5), with *P < 0.05 by student's *t-*test.

As knockdown of Ku80 suppressed the expression of PDK1 (Fig. 4) and melanoma growth (Fig. 2), we hypothesized that Ku80 regulated melanoma growth through PDK1. To test this hypothesis, we investigated whether overexpression of PDK1 could rescue the inhibitory effects caused by Ku80 knockdown in melanoma cells. MTS assay showed that knockdown of Ku80 inhibited the viability of A375 and MEWO cells, which was partially reverted by overexpression of PDK1 (Fig. 6A). In the same way, colony formation assay confirmed that overexpression of PDK1 partially reversed the decreased colony formation capacity by Ku80 downregulation in A375 and MEWO cells (Fig. 6B).

To further demonstrate that PDK1 mediated Ku80-regulated melanoma growth *in vivo*, we established a melanoma xenograft mouse model. In accordance with our *in vitro* study, Ku80 overexpression dramatically promoted melanoma growth in tumor volume, size and weight (Fig. 6C), while Ku80 knockdown effectively suppressed melanoma growth (Fig. 6D), and such an inhibitory effect was reverted in part by PDK1 overexpression (Fig. 6E). Altogether, these experiments supported that PDK1 was involved in the Ku80-regulated melanoma growth.

### 3.4 Ku80 interacts with HIF1-α to regulate PDK1 transcription

**Fig. 7:**
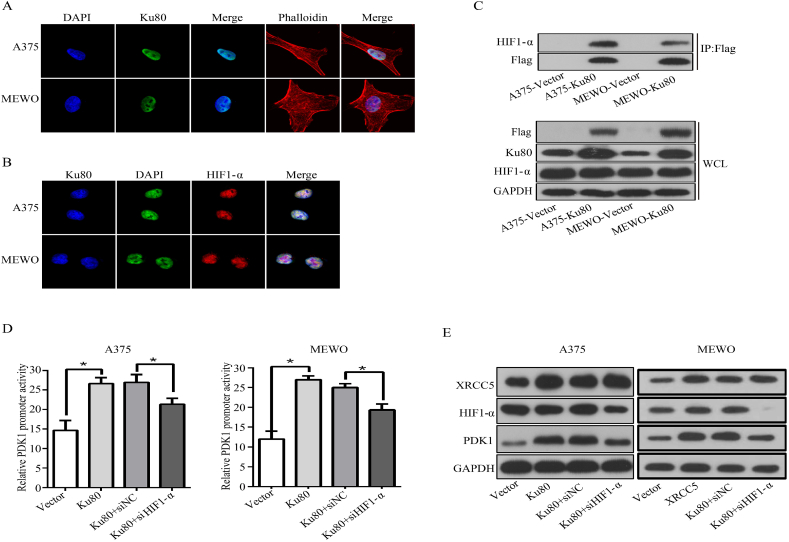
**Ku80 interacted with HIF-α and regulated the PDK1 expression. (A)** A375 and MEWO cells were cultured on chamber slides for 24 h, and immunofluorescence was applied to examine the subcellular localization of Ku80. The nuclei were stained by DAPI, Ku80 was detected by its specific antibody, and the cytoskeleton was labeled with rhodamine phalloidin for confocal microscopy. **(B)** A375 and MEWO cells were cultured on chamber slides for 24 h, and immunofluorescence was applied to examine the subcellular localization of Ku80 and HIF1-α. The nuclei were stained by DAPI, while Ku80 and HIF1-α were detected by their specific antibodies for confocal microscopy. **(C)** Protein extracts from A375 and MEWO cells stably overexpressing Flag-tagged Ku80 were immunoprecipitated with Flag antibody, and then the precipitates (IP, upper panel) as well as the whole cell lysates (WCL, lower panel) were analyzed by Western blot. **(D**–**E)** A375 and MEWO cells were transfected respectively with control vector (Vector), Ku80 overexpression plasmid (Ku80), Ku80 overexpression plasmid + control siRNA (Ku80+siNC), and Ku80 overexpression plasmid + HIF1-α siRNA (Ku80+siHIF1-α). After 72 h, relative PDK1 promoter activity was determined by dual-luciferase assay **(D)**, and the protein level of PDK1 was detected by Western blot **(E)**. The results were presented as mean ± SD of three independent experiments, with *P < 0.05 by student's *t-*test.

It has been reported that HIF1-α is a common transcription factor important for the expression of the PDK protein family, which includes PDK1, PDK2, PDK3 and PDK4 [41]. Accordingly, we speculated that HIF1-α might recruit Ku80 and facilitate its association with the PDK1 promoter. To examine this possibility, immunofluorescence was performed to detect the localization of Ku80 and HIF1-α in melanoma cells. The results demonstrated that Ku80 was localized to the nuclei of A375 and MEWO cells (Fig. 7A), and the localization of HIF1-α was also restricted to the nuclei of the cells (Fig. 7B), implying that Ku80 and HIF1-α had nuclear colocalization. To prove that Ku80 interacted with HIF1-α, co-IP experiment was done and showed that HIF1-α was detected in the protein complexes pulled down by Flag-tagged Ku80 (Fig. 7C). Furthermore, dual luciferase reporter assay revealed that the elevated PDK1 promoter activity contributed by Ku80 overexpression in A375 and MEWO cells was partially abolished by HIF1-α knockdown (Fig. 7D). Consistently, the protein level of PDK1 was increased in melanoma cells with Ku80 overexpression, which was reverted in part by knockdown of HIF1-α (Fig. 7E). Altogether, these results implicated that Ku80 interacted with HIF1-α in the regulation of PDK1 transcription.

### 3.5 Ku80 regulated the sensitivity of melanoma cells to melatonin

**Fig. 8:**
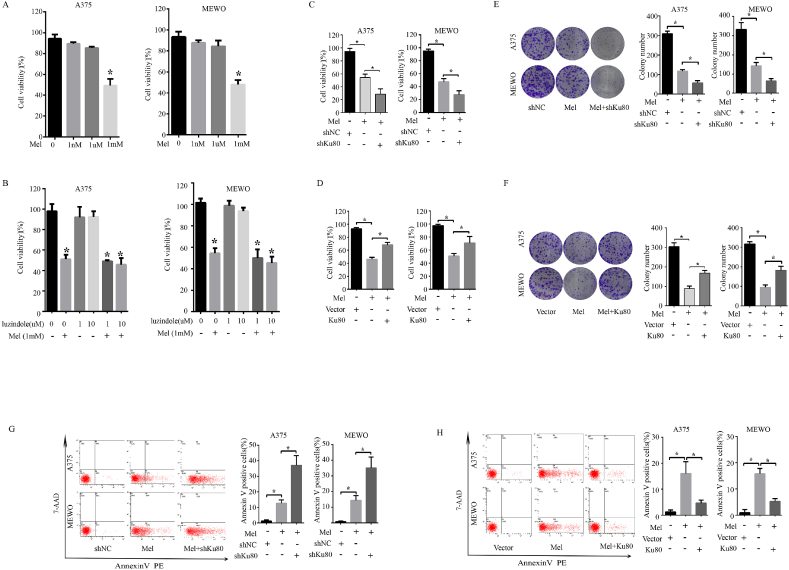
**Ku80 regulated the sensitivity of melanoma cells to melatonin *in vitro*. (A)** A375 cells or MEWO cells were incubated in presence of the indicated concentrations of melatonin for 72 h, Cell viability was measured by MTS assay. **(B)** A375 cells or MEWO cells were preincubated for 2 h with the indicated concentrations of melatonin antagonists luzindole and then incubated in presence of 1 mM melatonin for 72 h *P < 0.05, significantly different from melatonin untreated control. **(C)** A375 cells or MEWO cells were treated with 1 mM melatonin alone, or in combination with Ku80 shRNA (shKu80). Cells transfected with control shRNA (shNC) served as controls, Cell viability was measured by MTS assay. **(D)** A375 cells or MEWO cells were treated with 1 mM melatonin alone or Ku80 overexpression plasmid (Ku80) for assays. Cells transfected with control vector (Vector) served as controls, cell viability was measured by MTS assay. **(E)** A375 cells or MEWO cells were treated with 1 mM melatonin alone, or in combination with Ku80 shRNA (shKu80). Cells transfected with control shRNA (shNC) served as controls, 2 weeks later, colony formation capacity was determined by colony formation assay. **(F)** A375 cells or MEWO cells were treated with 1 mM melatonin alone or Ku80 overexpression plasmid (Ku80) for assays. Cells transfected with control vector (Vector) served as controls, 2 weeks later, colony formation capacity was determined by colony formation assay. **(G)** A375 cells or MEWO cells were treated with 1 mM melatonin alone, or in combination with Ku80 shRNA (shKu80). Cells transfected with control shRNA (shNC) served as controls. 72 h later, the cells were stained with Annexin V-FITC/PI, and their apoptosis was determined by FACS analysis. **(H)** A375 cells or MEWO cells were treated with 1 mM melatonin alone or Ku80 overexpression plasmid (Ku80) for assays. Cells transfected with control vector (Vector) served as controls. 72 h later, the cells were stained with Annexin V-FITC/PI, and their apoptosis was determined by FACS analysis. The results were presented as mean ± SD of three independent experiments, with *P < 0.05 by student's *t-*test.

**Fig. 9:**
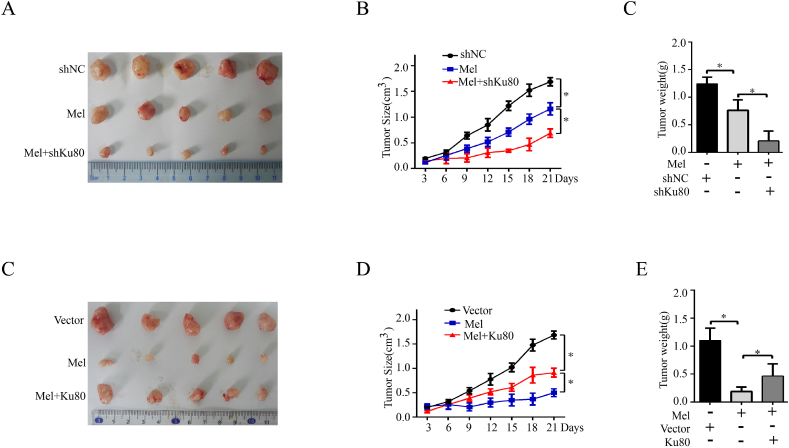
**Ku80 regulated the sensitivity of melanoma cells to melatonin *in vivo*. (A**–**C)** A375 stable cells with shNC or shKu80 were subcutaneously injected into the flank of the nude mice. Melatonin was injected intraperitoneally at a dose of 25 mg/kg once every two days. Tumor size was recorded every 3 days over 3 weeks (middle panels), and at the end of the experiment, the xenografts were excised after the mice were humanely sacrificed. Tumor volume was then captured (left panels), and tumor weight was measured (right panels). **(C**–**E)** A375 stable cells with Vector or Ku80 were subcutaneously injected into the flank of the nude mice. Melatonin was injected intraperitoneally at a dose of 25 mg/kg once every two days. Tumor size was recorded every 3 days over 3 weeks (middle panels), and at the end of the experiment, the xenografts were excised after the mice were humanely sacrificed. Tumor volume was then captured (left panels), and tumor weight was measured (right panels). The results were presented as mean ± SD (n = 5), with *P < 0.05 by student's *t-*test.

Melatonin has been demonstrated to have antitumor activity in a variety of cells both *in vitro* and in *vivo* [42], and a previous study has shown that melatonin inhibited the growth of Sk-mel-1 human melanoma cells at concentrations that exceeded its physiological levels in blood [43]. Moreover, a previous study has revealed that melatonin promotes cardiomyogenesis of embryonic stem cells via inhibition of HIF-1α stabilization [44]. As HIF1-α take part in the regulation of Ku80/PDK1 signal pathway which we have proved above. We propose that Ku80 may affect the sensitivity of melanoma cells to melatonin in HIF1-αdependent way, To prove this hypothesis, we firstly performed MTS and colony formation assays in melanoma cells treated with melatonin. As shown in Fig. 8A, treatment with melatonin at 1.0 mM significantly inhibited cell proliferation in A375 and MEWO cells, whereas treatment with melatonin at 1.0 nM or 1.0 μM showed slight inhibition. Therefore, we used melatonin at 1.0 mM for further study. However, the concentration of 1 mM melatonin is high which indicates that the cell lines do not synthesize melatonin and the blockage of melatonin receptors will not have a pro-proliferative effect. To examine whether the growth inhibitory effects of melatonin are receptor-mediated, experiments were performed in presence of a well characterized melatonin receptor antagonists (luzindole). The results indicated that the blockage of melatonin receptors did not show a pro-proliferative effect and suggest that cell growth inhibitory is independent of melatonin receptor. Our results then showed that knockdown of Ku80 remarkably enhanced the cell growth inhibition caused by melatonin, whereas overexpression of Ku80 partially reverted the antiproliferative effect of melatonin (Fig. 8C–F). Apoptosis is a process of programmed cell death which also influenced cell viability, and most anticancer drugs function primarily to induce apoptosis. We hypothesized that Ku80 potentiated the cytotoxicity of melatonin via the apoptosis signaling pathway. To test this hypothesis, we measured apoptosis of melanoma cells by flow cytometry. As showed in Fig. 8G, Ku80 knockdown potentiated melatonin-induced apoptosis compared to melatonin treatment alone. Inversely, overexpression of Ku80 decreased the number of apoptotic cells induced by melatonin in A375 and MEWO cells (Fig. 8H). Moreover, knockdown of Ku80 further suppressed the growth of human melanoma xenografts in nude mice when combined with melatonin treatment, while overexpression of Ku80 partially reversed the growth inhibitory effect of melatonin (Fig. 9A–E).

### 3.6 Melatonin synergizes with Ku80 knockdown to inhibit cells growth through HIF1-α dependent Ku80/PDK1 pathway

**Fig. 10:**
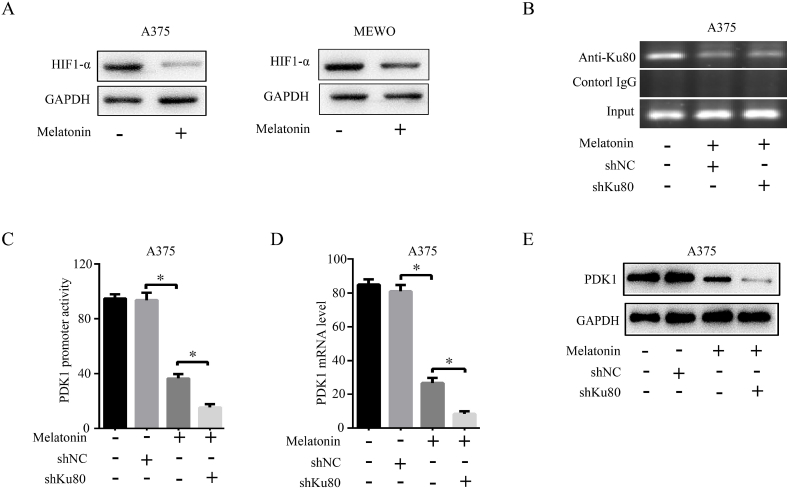
**Melatonin synergized with shKu80 to inhibit cells growth through HIF1-α dependent Ku80/PDK1 pathway. (A)** A375 cells or MEWO cells were treated with 1 mM melatonin for 72 h, the protein level of HIF1-α was detected by Western blot with its specific antibody. **(B)** A375 cells were treated with 1 mM melatonin or transfected with shKu80. After 72 h, ChIP was performed with Ku80 specific antibody, and the precipitated PDK1 promoter fragments were amplified by PCR using PDK1 promoter specific primers. The PCR products were separated in a 2% agarose gel. IgG was used as a negative control. **(C)** A375 cells were treated with 1 mM melatonin for 72 h, the mRNA level of PDK1 was detected by qPCR. **(D)** A375 cells were treated with 1 mM melatonin for 72 h, relative PDK1 promoter activity was determined by dual-luciferase assay. **(E)** A375 cells were treated with 1 mM melatonin alone or in combination with shKu80. After 72 h, the protein level of PDK1 was detected by Western blot with its specific antibody. The experiments were done in triplicates, and the results were presented as mean ± SD, with *P < 0.05 by student's t-test.

It had reported that melatonin could affect the stability of HIF1-α at the protein level, we proposed hypothesis that melatonin synergized with Ku80 knockdown through HIF1-α dependent Ku80/PDK1 pathway. Therefore, to gain insight into the molecular mechanisms of the synergistic interaction between shKu80 and melatonin, we detected the change of HIF1-α at the protein level, our study revealed that the HIF1-α was down-regulated with the treatment of melatonin (Fig. 10A). Additionally, ChiP assay showed melatonin suppressed the binding of Ku80 to the PDK1 promoter (Fig. 10B) and inhibited the activity of the PDK1 promoter (Fig. 10C), and then down-regulated the expression of PDK1 (Fig. 10D and E). This result suggested that the synergetic antiproliferative effect of melatonin and shKu80 through HIF1-α dependent Ku80/PDK1 pathway.

### 3.7 The expression of Ku80 and PDK1 were elevated and predicted poor prognosis in melanoma patients

**Fig. 11:**
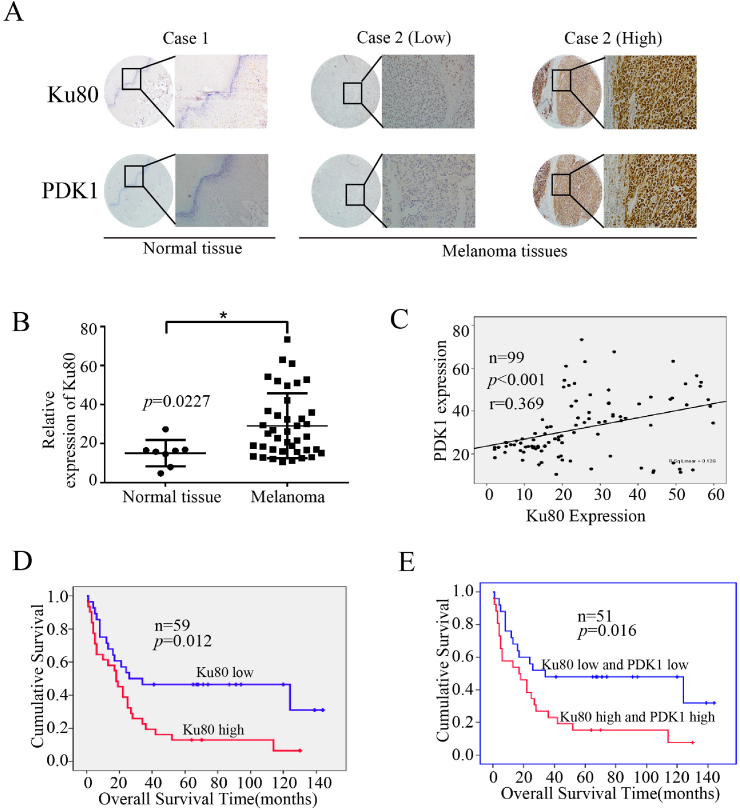
**Elevated expression of Ku80 and PDK1 predicted poor prognosis in melanoma patients. (A)** Representative images of the immunohistochemical staining of Ku80 and PDK1 in normal (left panel) and melanoma (middle and right panels) tissues. “Low” indicated low expression of both Ku80 and PDK1, while “high” indicated high expression of both proteins. **(B)** Quantification of Ku80 expression in normal and melanoma tissues detected by immunohistochemistry. The results were presented as mean ± SD, with *P < 0.05 by student's t-test. **(C)** Pearson correlation test was used to analyze the protein levels of PDK1 and Ku80 in melanoma tissues. **(D**–**E)** Kaplan-Meier overall survival curves were generated for melanoma patients with high or low expression of Ku80 **(D)** and patients with high or low levels of both Ku80 and PDK1 **(E)**.

To uncover the clinical significance of Ku80 and further confirm its relevance with PDK1, we detected their expression by immunohistochemical (IHC) staining in a tissue microarray that contained 99 melanoma samples and 8 normal samples (Fig. 11A). Our analysis revealed that the expression of Ku80 was significantly elevated in melanoma tissues (Fig. 11B), and the protein levels of PDK1 and Ku80 were positively correlated in melanoma samples (Fig. 11C). We then determined the relevance of Ku80 expression to various clinical features, and found that the expression of Ku80 was unrelated to patient gender and age (Table 1). However, analysis of another tissue microarray containing 59 melanoma samples with detailed survival information showed that high expression of Ku80 was associated with poor prognosis in melanoma patients (Fig. 11D), and patients with high levels of both Ku80 and PDK1 had worse prognosis than those with low expression of Ku80 and PDK1 (Fig. 11E). Collectively, our clinical data validated the oncogenic role of the Ku80/PDK1 pathway in melanoma.

**Table 1:** Correlation analyses of Ku80 expression in relation to the gender and age of melanoma patients.

Clinical Factor	XRCC5 Expression		*P*
	High	Low	
Gender			
Male	35 (59.3)	24 (40.7)	0.1
Female	17 (42.5)	23 (57.5)	
Age			
<55	23 (48.9)	24 (50.1)	0.143
≥55	30 (57.7)	22 (42.3)	

## 4 Discussion

Melanoma is a highly malignant tumor with very poor prognosis. Although the application of targeted therapies and immunotherapy has significantly improved the overall survival of patients, the prognosis is still not so favorable. Also, the development of drug resistance after a period of treatment has been an unneglectable problem. Therefore, it is still urgent to investigate the mechanism of melanoma development, and seek for potential therapeutic targets.

In this study, we screened a siRNA library in A375 melanoma cells to identify the genes that affected cell viability, and found that knockdown of Ku80 significantly suppressed the growth of melanoma cells. Ku80 is best known for its function in DNA damage repair in its heterodimer with Ku70 [20], while accumulating evidence has suggested that Ku80 also plays important roles in cellular processes related to cancer development, such as telomere maintenance and cell cycle regulation [[21], [22], [23]]. Our results showed that Ku80 knockdown inhibited the colony formation and viability of melanoma cells and promoted apoptosis. In contrast, overexpression of Ku80 enhanced the viability, colony formation of melanoma cells. Additionally, we found that down-regulation of Ku80 inhibited the PI3K/Akt/mTOR signaling pathway and activated the caspase/PARP pathway, whereas up-regulation of Ku80 activated the PI3K/Akt/mTOR signaling pathway. Our findings are in accordance with earlier studies that reported overexpression of Ku80 enhanced cell proliferation, and suppression of Ku80 induced apoptosis and blocked invasion in a variety of cancer cells [31,45].

Mechanistically, we found that Ku80 regulated the expression of PDK1 by binding to its promoter as a transcription factor. Knockdown of Ku80 down-regulated the transcription of PDK1, whereas overexpression of Ku80 up-regulated the transcription of PDK1. PDK1 is a member of the pyruvate dehydrogenase kinase (PDK) family that also includes PDK2, PDK3 and PDK4 [41]. PDK1 is an important metabolic enzyme in aerobic glycolysis [38,46], and highly expressed in many tumors including melanoma [39,40]. Consistently, our results showed that the expression of Ku80 and PDK1 were positively correlated in melanoma tissues.

Previous studies have reported that the Ku heterodimer could generally displace transcription factors when bound to DNA and thus inhibit transcription [25], or specifically regulate target genes expression through its interaction with other transcription factors [24]. Considering that PDK1 has been found to be activated by the transcription factor HIF-1 (hypoxia-inducible factor 1) [38,47], we therefore speculate that Ku80 could be recruited to the promoter of PDK1 by HIF-1, which requires future investigation. Also, it is worth further examination whether Ku80 regulates the transcription of PDK1 independently, or by forming the Ku heterodimer with Ku70, for it has been suggested that individual Ku subunits are unstable [20].

Melatonin is a hormone mainly produced by the pineal gland. It has been found to be a pleiotropic molecule that functions in gene expression regulation, anti-proliferation, immunity modulation, anti-inflammation, anti-oxidation and anti-angiogenesis, and its effects for cancer management have been under clinical investigation [41]. Previous studies have shown that melatonin inhibited human melanoma cells growth at concentrations that exceeded its physiological levels in blood [43]. Other studies showed the proapoptotic effects of melatonin on tumor cells [48] while antiapoptotic effects in non-tumor normal cells [49,50]. And the synergistic effects of melatonin and chemotherapy on apoptosis in tumor cells had been reported [51,52]. Moreover, another study has revealed that melatonin promotes cardiomyogenesis of embryonic stem cells via inhibition of HIF-1α stabilization [44]. In this study, we found that down-regulation of Ku80 enhanced the antitumor effect of melatonin in melanoma. Mechanistically, we found that the synergistic antitumor effect of melatonin and Ku80 knockdown was mediated by HIF1-α. So far, a few strategies have been developed to specifically target the Ku complex [20]. For example, HNI-38 is a synthesized peptide representing the C terminus of Ku80 that can selectively disrupt the interaction between Ku and DNA, and thus reduce its activity in double-stranded DNA break repair, and it has been shown to potentiate the effect of ionizing radiation in breast cancer cells [53]. It is intriguing to test if HNI-38 can also inhibit the Ku80-regulated PDK1 expression, and synergize the oncostatic effect of melatonin, indicating that melatonin and Ku80 inhibitors in combination might be useful for melanoma treatment.

Our analysis of clinical samples showed that high expression of Ku80 and PDK1 was associated with poor prognosis in melanoma patients. This is in consistence with the findings by Song et al. that the expression of Ku80 increased as melanoma developed from benign naevi to different stages of primary and metastatic tumors, and was inversely associated with favorable survival of patients [33]. It is currently unclear the mechanism underlying the elevated expression of Ku80 during melanomagenesis. As genome instability is a hallmark of cancer, it is possible that accumulating DNA damages due to the active DNA synthesis in cancer cells activate certain signaling pathways to increase the expression of DNA repair proteins like Ku80 to maintain genome integrity [32]. On the other hand, NHEJ is intrinsically error-prone as it directly religates the broken DNA ends [54], and overexpression of its major player Ku80 may allow cancerous cells to survive their many DNA damages, resist chemotherapy and radiotherapy, and mutate to acquire the capacity to metastasize.

**Fig. 12:**
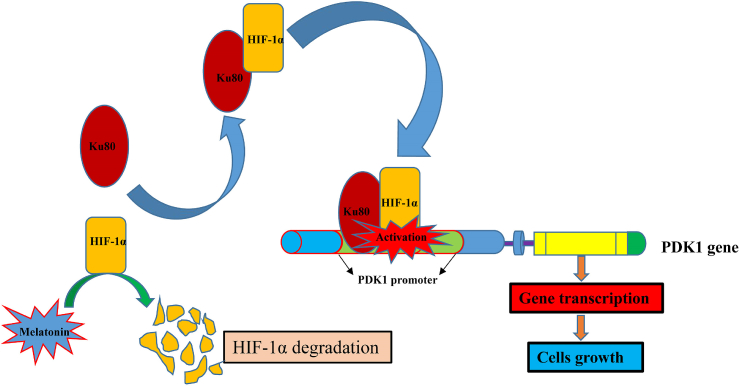
**Schematic model: Ku80 promotes tumor growth and regulates sensitivity to melatonin via PDK-1 signaling pathway in melanoma cells**. Ku80 bound to the promoter of PDK1 to enhance its transcription was HIF1-α dependent. Melatonin promoted the degradation of HIF1-α, thus weakening the combination of Ku80 with PDK1 promoter.

In summary, our study found that Ku80 bound to the promoter of PDK1 to enhance its transcription via a HIF1-α dependent manner. Melatonin promoted the degradation of HIF1-α, thus weakening the combination of Ku80 with PDK1 promoter (Fig. 12). Furthermore, Ku80 and PDK1 were highly expressed in melanoma tissues, and associated with poor prognosis in melanoma patients. These findings have suggested that Ku80 may be a potential target and melatonin combines with Ku80 inhibitor may be a more powerful treatment mode for melanoma treatment.

## Conflicts of interest

The authors declare no potential conflicts of interest.

## Author contributions

This work was carried out in collaboration between all authors. TL, TK and WD defined the research theme and designed the experimental approach. TL, LJ, MC, ZZ, WL, WF, LL, FZ, QZ, HQ, JL, MC, CT, ZH, CZ, YL carried out the experiments. TL, ML JL, LY, YL and WD analyzed the data and interpreted the results. TL, MC and WD wrote the manuscript.
